# A blueprint for patient and public involvement in the development of a reporting guideline for systematic reviews of outcome measurement instruments: PRISMA-COSMIN for OMIs 2024

**DOI:** 10.1186/s40900-024-00563-5

**Published:** 2024-03-21

**Authors:** Ellen B. M. Elsman, Maureen Smith, Catherine Hofstetter, Frank Gavin, Estelle Jobson, Sarah Markham, Juanna Ricketts, Ami Baba, Nancy J. Butcher, Martin Offringa

**Affiliations:** 1 Child Health Evaluative Sciences, The Hospital for Sick Children Research Institute, Toronto, ON Canada; 2 Amsterdam UMC, Vrije Universiteit Amsterdam, Amsterdam Public Health Research Institute, Epidemiology & Data Science, Amsterdam, Netherlands; 3 Patient Partner, Cochrane Consumer Network, Ottawa, ON Canada; 4 Patient and Public Contributor/Advisor, OMERACT Patient Research Partner, Toronto, ON Canada; 5 Patient and Public Contributor, Chair of the Public Advisory Council of The Health Data Research Network (Canada), Toronto, ON Canada; 6 Patient and Public Contributor, EUPATI Fellow, Nyon, Switzerland; 7 Patient and Public Contributor, King’s College London, London, UK; 8 Patient and Public Contributor, Halifax, NS Canada; 9 Department of Psychiatry, University of Toronto, Toronto, ON Canada; 10 Institute of Health Policy, Management and Evaluation, University of Toronto, Toronto, ON Canada; 11 Peter Gilgan Centre for Research and Learning, The Hospital for Sick Children, 686 Bay Street, Toronto, ON M5G 0A4 Canada

**Keywords:** Patient and public involvement (PPI), Patient engagement, Reporting guideline, Systematic reviews, Outcome measurement instrument, PRISMA, COSMIN

## Abstract

**Background:**

In recent years, projects to develop reporting guidelines have attempted to integrate the perspectives of patients and public members. Best practices for patient and public involvement (PPI) in such projects have not yet been established. We recently developed an extension of PRISMA (Preferred Reporting Items for Systematic Reviews and Meta-Analyses), to be used for systematic reviews of outcome measurement instruments (OMIs): PRISMA-COSMIN (COnsensus-based Standards for the selection of health Measurement INstruments) for OMIs 2024. Patients and public members formed a small but impactful stakeholder group. We critically evaluated the PPI component in this project and developed recommendations for conducting PPI when developing reporting guidelines.

**Main text:**

A patient partner was an integral research team member at the project development and grant application stage. Once the project started, five patient and public contributors (PPCs) were recruited to participate in the Delphi study; three PPCs contributed to subsequent steps. We collected quantitative feedback through surveys; qualitative feedback was garnered through a focus group discussion after the Delphi study and through debrief meetings after subsequent project activities. Feedback was thematically combined with reflections from the research team, and was predominantly positive. The following themes emerged: importance of PPI partnership, number of PPCs involved, onboarding, design of Delphi surveys, flexibility in the process, complexity of PPI in methodological research, and power imbalances. Impacts of PPI on the content and presentation of the reporting guideline were evident, and reciprocal learning between PPCs and the research team occurred throughout the project. Lessons learned were translated into 17 recommendations for future projects.

**Conclusion:**

Integrating PPI in the development of PRISMA-COSMIN for OMIs 2024 was feasible and considered valuable by PPCs and the research team. Our approach can be applied by others wishing to integrate PPI in developing reporting guidelines.

**Supplementary Information:**

The online version contains supplementary material available at 10.1186/s40900-024-00563-5.

## Abstract (highlights)

Reporting guidelines help authors in reporting a specific type of research. They are often presented in the form of a checklist. In recent years, efforts have been made to include the perspectives of patients and members of the public to make sure that what matters to them is also reported. However, best practices for patient and public involvement in such projects do not yet exist. We developed a new reporting guideline for systematic literature reviews of outcome measurement instruments, called “PRISMA-COSMIN for OMIs”. In the development of this guideline, one patient partnered as a member of the research team, and five other patients and public members participated in the project. They all had a positive impact on the content and presentation of the guideline. We invited patients and public members to reflect and give feedback on their involvement in the project. Reflections and feedback were mostly positive. Key strengths of our approach were:

1. Limiting power imbalances by having a patient on the research team.
2. Organizing a preparatory meeting for patients and public members to go over key project details and concepts.
3. Remaining flexible in how patients and public members could contribute.

This project shows that our approach is feasible. Here, we share insights we gained throughout the project. We translate “lessons learned” into 17 recommendations. These recommendations can be used by researchers who wish to include patient and public perspectives in developing new reporting guidelines.

**Supplementary Information:**

The online version contains supplementary material available at 10.1186/s40900-024-00563-5.

## Introduction

A research reporting guideline is a checklist, flow diagram, or structured text that guides authors in reporting a specific type of research, developed using explicit methodology. The Enhancing the QUAlity and Transparency Of health Research (EQUATOR) Network collects and provides reporting guidelines to achieve accurate, complete, and transparent reporting of all health research to make it reproducible and useful [1]. Specific reporting guidelines exist for different health research types, and updates, extensions, and new reporting guidelines are continually being developed and published [2].

Recently, there has been interest in integrating patient and public involvement (PPI) in developing reporting guidelines by including patients and public members as stakeholders [3–5]. For example, in the SPIRIT- and CONSORT-Outcomes extensions, patients and public members were included as stakeholders, but directly before and during the consensus meeting [4, 5]. The integration of patient and public perspectives on what items should be reported in health research is important since members of these overlapping groups will be most impacted by the outcomes of health research. Including patients and public members as stakeholders enables researchers to improve reporting on matters that patients and public members deem important. Although EQUATOR has identified patients and public members as a stakeholder group to include when developing a reporting guideline [6], no established guidance or “best practices” on how to engage and involve patients and public members in the development of reporting guidelines are available. A recent study found that out of 262 reporting guidelines assessed, only 9 included patient/public representatives at the consensus meeting [7].

We recently developed an extension of the PRISMA (Preferred Reporting Items for Systematic Reviews and Meta-Analyses) guideline [8], so that it can be used by authors reporting systematic reviews of outcome measurement instruments (OMIs). Such systematic reviews synthesize the findings of individual studies on the measurement properties of OMIs and inform decisions about using OMIs in prospective clinical research and in practice [9]. This new reporting guideline is called PRISMA-COSMIN (COnsensus-based Standards for the selection of health Measurement INstruments) for OMIs 2024 [10] and has been developed following the approach recommended by EQUATOR [11].

In the development of PRISMA-COSMIN for OMIs 2024, patients and public members formed an impactful stakeholder group. Throughout the development of PRISMA-COSMIN for OMIs 2024, we carefully planned and evaluated the PPI component of the project, i.e., the roles and activities of patients and public members during each phase of the guidance development to provide concrete input into the end product. This offered important insights we wish to share. In this communication, we first describe how we partnered with patients and public members in developing the reporting guideline and adapted our strategy as the project evolved. We then reflect on lessons learned from the PPI strategy we followed. We use the GRIPP2-short form (Guidance for Reporting Involvement of Patients and the Public) [12] to report on PPI. Reporting guideline developers who wish to establish effective and meaningful PPI may use the information in this communication while planning and designing their PPI component.

## Main text

### PPI in PRISMA-COSMIN for OMIs 2024

**Fig. 1:**
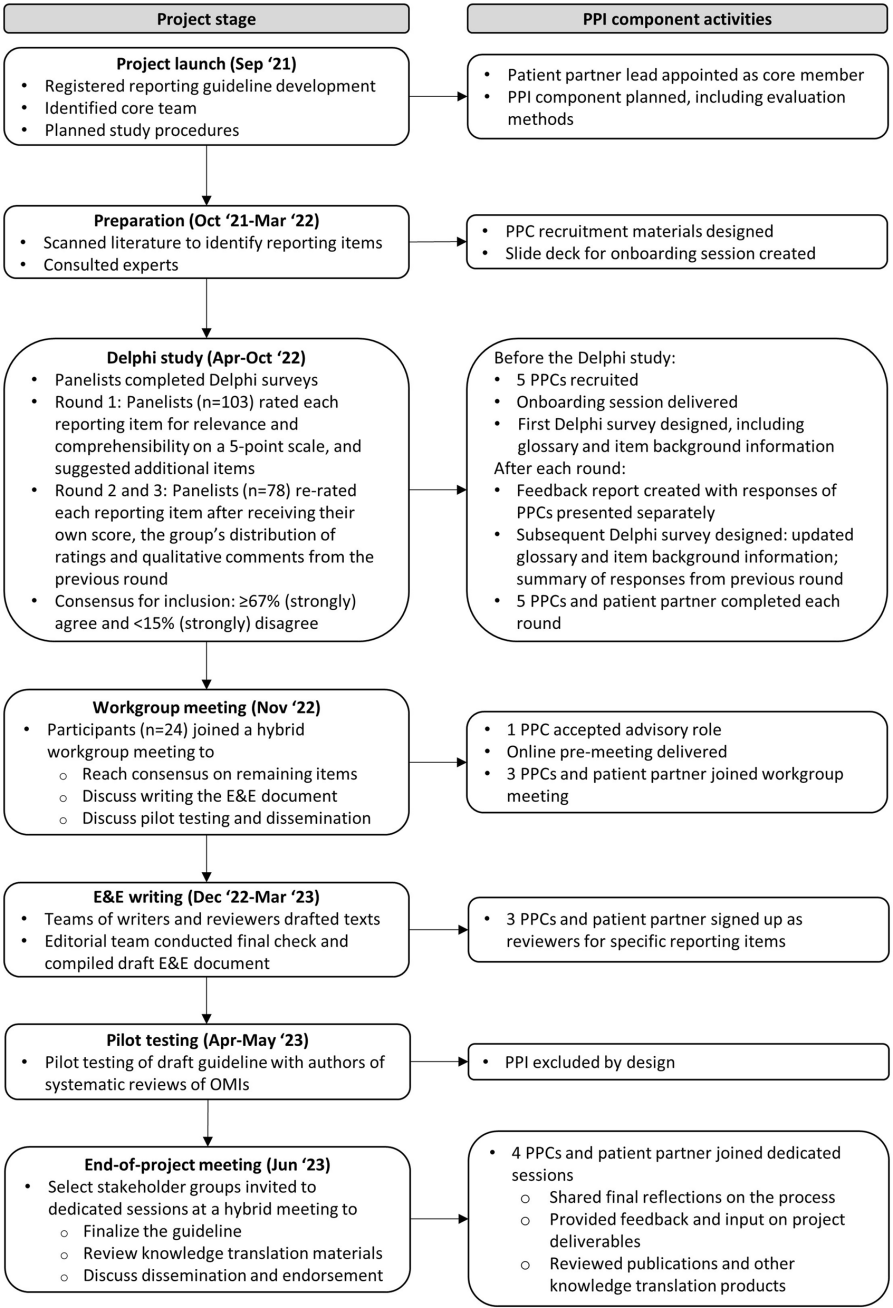
PPI component activities at each step in the PRISMA-COSMIN for OMIs’ guideline development. E&E, Explanation and Elaboration; OMIs, outcome measurement instruments; PPI, patient and public involvement; PPCs, patient and public contributors

Figure 1 outlines the seven stages in the development of PRISMA-COSMIN for OMIs 2024 and presents the outputs relevant to PPI. A protocol has been published elsewhere [10]; final results of this process are published elsewhere as well [13]. Briefly, the project included an environmental scan of the literature, a Delphi study, and a workgroup meeting to arrive at a consensus-based draft checklist of reporting items. This reporting checklist was subsequently pilot tested and texts for the Explanation & Elaboration (E&E) document were drafted using a group writing process. During an end-of-project meeting the reporting guideline, which includes the checklist, E&E, and flow diagram, was finalized. Here we describe the methods specific to PPI.

An experienced patient partner (MS) who was able to contribute at a co-investigator level and co-lead the PPI component was recruited as a core member at the grant application stage. This patient partner was actively engaged in steering committee meetings throughout the entire project and provided input at key stages. Once the project started, five patients and public members were recruited through newsletters and contact persons of relevant organizations (Cochrane Consumer Network [14], SPOR Evidence Alliance [15], COMET [16], and OMERACT [17]) to participate in the Delphi study. Most of them had experience with being patient research partners (in other projects) and have previously published patient reflections [18–20]. We refer to them as patient and public contributors (PPCs) in the remainder of this communication, as their role was different from the patient partner, who was a core member of the research team from the start of the project.

Prior to the Delphi study, an online onboarding session was delivered and led by the patient partner and the postdoctoral research fellow. The aim of the onboarding session was twofold: 1) to provide the PPCs with a fundamental understanding of the project objectives and methods and review essential concepts such as systematic reviews, outcome measurement instruments, and reporting guidelines; and 2) to discuss the rationale for including PPCs and why their perspectives were important in selecting what should be reported.

During a focus group discussion after the Delphi study, PPCs were offered the opportunity to contribute to subsequent steps of the reporting guideline development process. Three PPCs accepted and joined a hybrid workgroup meeting with other stakeholders to discuss and reach agreement on the inclusion, exclusion, and wording of items that did not reach consensus during the Delphi study. As the opportunity for this level of involvement at this stage of the project arose organically, one of the PPCs who had expertise with similar meetings accepted an advisory role and participated in the planning and organization of the hybrid workgroup meeting. An online pre-meeting was organized to review workgroup meeting materials and procedures with PPCs.

Following the workgroup meeting, texts for the E&E document were drafted using a group writing process. The three PPCs who attended the workgroup meeting signed up to be reviewers for reporting items, including specific items that would benefit greatly from patient and public input.

In the final stages of the project, four PPCs attended dedicated sessions at–and the week after–the end-of-project meeting and shared their final reflections on the PPI process and provided feedback on the project deliverables.

The main impact of PPCs contributions were the inclusion of three reporting items related to (1) feasibility and interpretability aspects of the OMI, (2) recommendations on which OMI (not) to use, and (3) the plain language summary. While other Delphi panelists often saw little relevance for these three items in the first Delphi round, PPCs felt strongly about their inclusion. PPCs arguments ultimately persuaded other Delphi panelists, and these items were voted into the final reporting checklist. PPCs led the rewording of reporting items and E&E text, resulting in a clearer guideline.

Throughout the project, the patient partner and PPCs were compensated for their time and contributions. The patient partner received honoraria as a co-investigator, whereas PPCs received gift cards after each project component they contributed to in accordance with the Canadian Institutes of Health Research (CIHR) Strategy for Patient-Oriented Research (SPOR) guidelines for compensation [21]. The patient partner and PPCs are co-authors of this manuscript.

#### Evaluating PPI

PPCs completed evaluation surveys after the onboarding session and Delphi study. The content of existing evaluation tools was often not applicable to our project [22–25]; therefore we modified their content to align with project activities. After the onboarding sessions, they completed modified versions of the Acceptability E-Scale [22] and the Public and Patient Engagement Evaluation Tool (PPEET) [23, 24]. After the Delphi study, they completed modified versions of the Acceptability E-Scale, the Patient Engagement In Research Scale (PEIRS) [25] and the PANELVIEW tool [26]. Within three weeks of completion of the Delphi study, an online focus group discussion with PPCs was conducted to obtain additional qualitative feedback. The focus group discussion was recorded and transcribed verbatim to obtain illustrative quotes. An online debrief meeting was held after the workgroup meeting, and notes regarding positive and negative experiences were collected. During the E&E writing process, the PPCs stayed connected as a group and shared their positive and negative experiences afterwards. Four PPCs joined a dedicated PPI session at the end-of-project meeting to reflect on the PPI component of the project.

### Summary of reflections and feedback

Feedback from the evaluation surveys administered after the onboarding session and Delphi study can be found in Additional files 1 and 2. Here, we thematically combine our own reflections with the feedback obtained from the evaluation activities.

#### Overall experience

Reflections on the overall experience were mostly positive. The research team and PPCs learned from one another throughout the process and thought this project could function as a blueprint for future research projects wishing to integrate PPI:

*“I imagine this is quite a rare situation to have something so technical being assisted with patient input. […] What you've done in a sense in this process is giving us a kind of a training experience, to learn how to do something, and that in itself is just sort of a contribution to the whole field of patient and public involvement. And that's why I said maybe you could come up with some sort of a blueprint. […] The fact that you did this in a very thoughtful way and that we all stayed with it, that's really telling. It means that you did good, and you gave us the chance to learn something new as well.”*

#### Importance of PPI

The research team recognized the importance of PPI in developing a reporting guideline for systematic reviews of OMIs, as patients and public members are ultimately impacted by the results of these systematic reviews. Based on these reviews, OMIs are selected to conduct measurements in research and clinical practice. PPCs shared this opinion, and one PPC commented:

*“Whether we [patients and public members] are actually using this reporting guideline or not doesn't really matter. It's important that we are able to give our input through our perspectives and our lived experience as to what is important to us to see reported in guidelines and in a systematic review.”*

PPCs recognized the contributions they made to the project. They thought it was empowering that PPCs often were on the same page about inclusion of certain items, for example for the reporting items related to feasibility and interpretability aspects of the OMI, recommendations on which OMI (not) to use, and the plain language summary.

*“We were seeming all to see something was particularly important to include, that the rest of the people weren't seeing as nearly as important. So, I think that kind of validated it and I certainly had a real strong sense that those elements, I really thought: Yes, we were all pretty much on the same page, and that strong advice, or guidance, wouldn't have come through if there weren't, you know, half a dozen people like us.”*

#### Number of PPCs involved

By design, only a small number of PPCs were recruited to participate in the Delphi study, as PPI in reporting guideline development is still in its infancy and we wanted to involve a small group and conduct robust evaluations of the PPI component. Budget allocation also informed the decision to recruit a limited number of PPCs. PPCs realized it is not always possible to involve more people, although involving more PPCs might offer different perspectives.

*“I always like seeing more patients involved because the diversity of perspectives and lived experiences, even from one person and globally too, from what your experiences are due to the access to healthcare and all kinds of other issues. The more patients you have, the broader range that you’re going to get of perspectives and experiences. So I’d love to see more patients all the time, but that just isn’t always feasible.”*

They also mentioned how recruiting more PPCs for the Delphi study might result in more PPCs being involved in later project stages, as some PPCs might decide not to continue with subsequent project activities, leaving only a limited number of PPCs who could contribute to, for example, drafting and reviewing the E&E document.

Although we made specific efforts, we were not successful in recruiting PPCs from lower- and middle-income countries. This may also have been due to the recruitment approach being very targeted; we did not have an extensive recruitment campaign or open call on social media platforms inviting everyone to participate in the project. The relative lack of diversity and other options for recruitment strategies were also discussed.

*“I feel that there should be more diversity and not just diversity as in gender, or as in low income, marginalized, disabilities, seniors. All the above and others make up the population, so how can we give our voices and accurate information if we don't have diversity for the whole, or at least a portion of the population? So I definitely would want to see it for everybody. To have the option. It is certainly up to that person if they want to or not, but it should be for everybody.”*

#### Onboarding session

As it was anticipated that PPCs may not have participated in methodological research before and that the purpose of the project (developing a reporting guideline for systematic reviews of OMIs) was likely distant from their immediate lived experience, an onboarding session was organized. We believe that this onboarding session contributed greatly to PPCs’ understanding of the project and increased their enthusiasm to undertake the project together. The onboarding session was greatly valued by the PPCs.

*“I was kind of skeptical about the onboarding session because I know about Delphi's, I've done them before. Blah, blah, blah. Then, it was very eye-opening, it was very helpful, it was very informative. It really set the stage for what we were about to do.”*

*“I think it was nice to see other people, get the feeling that you are part of a group. Even though the onboarding time is very limited, it still connects you.”*

#### Design of Delphi surveys

As the study topic was probably unfamiliar to the PPCs, a glossary was created that could be downloaded at each point in the Delphi survey to explain important concepts. Moreover, for each reporting item proposed in the Delphi study, background information was provided detailing the rationale for the item and, if possible, some examples. Both these resources, co-developed with the patient partner, helped PPCs understand the sometimes-technical reporting items. However, some PPCs thought the Delphi surveys were not always easy to complete and found the content of reporting items difficult to understand. The objectives and information about the process were rated as unclear by some PPCs as well. PPCs shared that they found the first round very difficult to understand but that their understanding improved over the subsequent rounds.

*“I found the wording really difficult to claw through, the first time. I'm used to the glossary and the explanations; I think that's an excellent thing that the Delphi has. But I found the wording really difficult to weed through and so, it took me quite a bit longer to do the first round. […] But then the second and third rounds were much, much easier to do and took much less time.”*

We believe that updating the glossary, background information and reporting items after each round based on the comments and suggestions of panelists, especially the PPCs, resulted in improved understanding of PPCs. In the Delphi surveys, the option to select “not my expertise” was included if panelists did not want to vote on the inclusion/exclusion or clarity of wording for an item. Although this option was available for all panelists, the research team anticipated this option to be particularly useful for PPCs. PPCs made good use of this option in the first round, but in the second and third round hardly ever used this option, most likely because of the improved wording of items and information.

After each Delphi round, a feedback report was created in which the distribution of responses from all panelists was presented, and for various stakeholder groups separately, in which PPCs formed one group. Detailed qualitative (anonymous) comments were also presented separately for each stakeholder group. A summary of the comments was provided in each subsequent Delphi study, so that panelists did not necessarily have to consult the extensive and detailed feedback report. If a difference in responses was noted between various stakeholder groups, this was presented in the summary of the comments, a feature valued by PPCs.

*“One thing I found very interesting and helpful was that when we got to round 2 and 3, there would be a comment that all the patients, or both of the patients all thought this element was quite important. As distinct from the other people who were doing the Delphi. So that was interesting. You think, well, there is real value here.”*

#### Flexibility in the process

The original intent of PPI in our project was to integrate this stakeholder group as contributors to the Delphi study and potentially invite them to participate in the consensus meeting. As the project evolved and changes were made to the process, PPCs were offered the opportunity to participate in a workgroup meeting that would lead to a collaboration in drafting the E&E document. One of the PPCs who had expertise with similar meetings accepted an advisory role and joined the steering committee to advise on this process. When PPCs were invited, we stressed the value of their contributions to the Delphi study and ensured that this was an opportunity and not an additional commitment. Three of five PCPs opted to be involved at this additional stage. They appreciated the fact that it was optional and that their involvement started with the Delphi study and along the way they were offered the possibility to be involved in subsequent phases. They mentioned how initially inviting them only for the Delphi study might have been the most optimal strategy, as it allowed them to commit to subsequent project activities on a case-by-case basis. Our flexible approach created opportunities for further and more in-depth collaboration with the PPCs and resulted in a reporting guideline for which the content was co-developed by patients and public members.

#### Complexity of PPI in methodological research projects

After the Delphi study, some PPCs disclosed that they were concerned that perhaps they focused too much on aspects that were not relevant to the project and were uncertain whether they completed the Delphi surveys correctly.

*“One thing I would have appreciated I suppose was after round 1 for example and I know I don't want to make extra work for anybody, but the whole idea was like: Am I doing something wrong? Or am I doing it some sort of... And I'm new at this so I'm quite happy to be told you're emphasizing this too much, or we would like to hear more about this but not about that, some sort of candid feedback. We’re all learning in this, so that would have helped me a little bit.”*

PPCs reported that completing the Delphi surveys took them longer than the durations that were estimated, which contributed to their lack of confidence.

*“Was I doing something wrong, and even though I got quite a bit of experience doing them, it was like: Am I doing something wrong that it's taking me longer than what they estimated for me to go through this.”*

The debrief meeting revealed that the PPCs at times felt overwhelmed at the workgroup meeting because other meeting attendees had high expectations about their role in drafting the E&E for the reporting guideline. During the E&E writing process, PPCs at times felt that they could not contribute to everything because the technicality of the written content was above their level of understanding. That did not dissuade them, ultimately, as they could give their feedback to specific paragraphs and examples and appreciated being part of the process.

#### Power imbalances

The patient partner co-led all PPI activities such as recruitment, communication, onboarding/pre-meetings, and evaluation sessions. More important, the patient partner shared her experiences as a contributor to Delphi surveys and consensus meetings in previous reporting guideline development projects, acknowledging the challenges for PPCs, and acted as an intermediary between researchers and PPCs. We believe that this mitigated power imbalances that may have existed if only a researcher had led these sessions.

*“I felt that I had my safe person. My go-to person, that I felt comfortable with. No matter what the questions or how I was feeling or wasn't feeling. I don't think I would have finished this if it had not been for (name patient partner), being able to reach out to her and talk to her. So, I feel that going forward it's something that these programs should have, is that contact person. Because she was really great in understanding and that safe person. Because for me, I'm kind of new to these specific projects, and I felt like a fish out of the water, and it was good to talk with her knowing that she is a person as well as myself and people with lived experience.”*

In the workgroup meeting, PPCs were part of a larger group of researchers, clinicians, and methodologists. It was important to the research team that PPCs were supported to participate fully and meaningfully in the workgroup meeting, without experiencing power imbalances. These power imbalances were mitigated by giving the floor to the patient partner to describe how the PPCs were involved in the project and by emphasizing the comments of PPCs in the discussion of the results.

## Recommendations

**Fig. 2:**
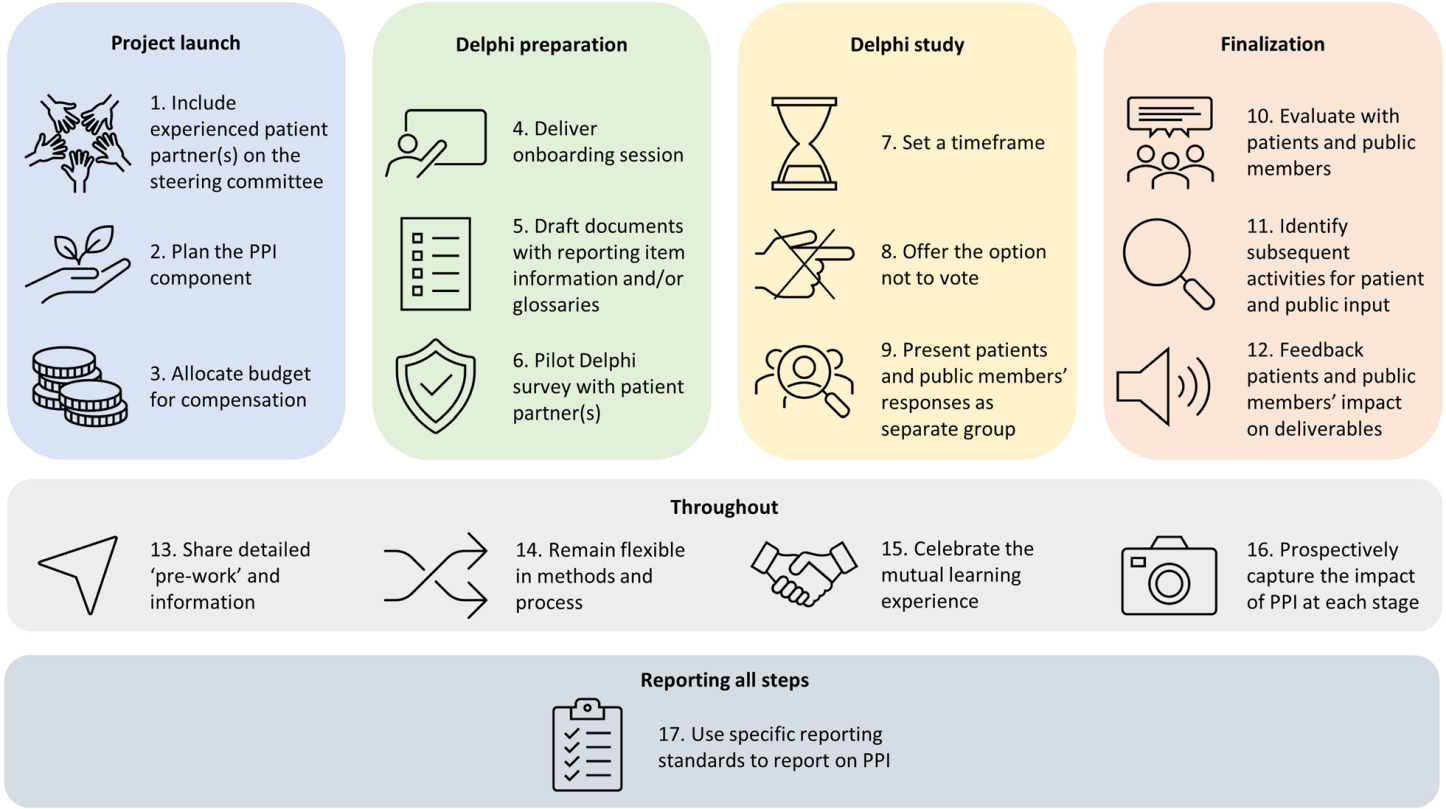
Blueprint: 17 steps to meaningful Patient and Public Involvement (PPI) in reporting guideline development

PPCs were keen for this project to serve as a blueprint for how to integrate PPI in the development of reporting guidelines. We turned “lessons learnt” from the PRISMA-COSMIN for OMIs PPI component into 17 recommendations for good PPI practice, summarized in Fig. 2. Table 1 offers more detailed guidance to other researchers who wish to integrate PPI in their efforts to develop reporting guidelines.

**Table 1:** Blueprint for meaningful Patient and Public Involvement (PPI) in reporting guideline development

Project stage	Recommendation
Project launch	1. *Include experienced patient partner(s) on the steering committee:* Invite the patient partner(s) to design and (co-) lead the PPI component to mitigate power imbalances and ensure relevance of project to the end-users 2. *Plan the PPI component:* Decide on (a) the number of patient and public members to involve, (b) how to recruit them, (c) when and how to involve them in the project, and (d) how to evaluate the PPI component of the project with both patients and public members, and with the research team 3. *Allocate budget for compensation:* Compensate patients and public members and the patient partner(s) for their time and contributions to various project stages in accordance with (inter)national guidelines, factoring in preparation time
Delphi study preparation	4. *Deliver onboarding session:* Review the project objectives and methods, essential concepts, why PPI is important, and be clear on the roles and expectations of patients and public members, and what patients and public members can expect in return. Ask the project team to join the first 10 min to introduce themselves and get to know the patients and public members. Allow time for questions during and after the session 5. *Draft documents with reporting item information and/or glossaries:* Provide background information for each reporting item and explain important concepts; pilot materials with patient partner(s); include background documents and glossaries in the Delphi survey; update this information after each Delphi round 6. *Pilot Delphi survey with patient partner(s):* Ask patient partner(s) to test Delphi surveys to ensure clarity on the objectives, methods, instructions, and wording
Delphi study	7. *Set a timeframe:* Provide an estimate of the time needed to complete the Delphi survey and reassure patients and public members that it is normal if it takes longer or shorter; highlight the possibility of completing the survey progressively in more than one sitting 8. *Offer the option not to vote:* Include a ‘not my expertise’ option and emphasize in the instructions that it is okay to select this option 9. *Present patients and public members’ responses as separate group:* Highlight responses of patients and public members if different from those of other stakeholders
Finalization	10. *Evaluate with patients and public members:* Besides planned evaluation methods, ask patients and public members if they have additional reflections on their involvement, and how they want to share their feedback 11. *Identify subsequent activities for patient and public input:* Be flexible and offer patients and public members opportunities to engage in subsequent project activities, even if PPI in subsequent stages was not planned for from the start; establish what is needed for meaningful involvement 12. *Feedback patients and public members’ impact on deliverables:* Report back on how the project deliverables have changed or developed because of their contributions
Throughout the project	13. *Share detailed ‘pre-work’ and information:* Ensure patient partner(s) co-writes or reviews all information and documents that will be sent to patients and public members 14. *Remain flexible in methods and process:* Listen to the needs and wishes of patients and public members; remain flexible and willing to adjust methods and process as the project evolves 15. *Celebrate the mutual learning experience:* Create an atmosphere of reciprocity, encouraging researchers and patients and public members to learn from one another, and offer feedback at each project stage, underscoring that all contributions are valuable 16. *Prospectively capture the impact of PPI at each stage:* Outline in a separate record the tangible and measurable changes stemming from PPI input during each stage of the project in real-time; disseminate/publish these changes in a knowledge translation piece
Reporting the project	17. *Use specific reporting standards to report on PPI:* Adhere to the GRIPP2 [12] reporting guideline to transparently and comprehensively account for PPI

### Strengths and weaknesses

Our approach has some key strengths. By including a patient partner as co-investigator as well as PPCs, we had varied levels of involvement across the International Association of Public Participation (IAP2) spectrum [27]. This resulted in opportunities for patients and public members who were new to PPI to contribute at various stages of the project. Our joint-learning approach [28, 29], being open towards changes in the project process [30] and transforming the role of one of the PPCs to be more advisory, ensured full and meaningful participation of all PPCs throughout the project. It also allowed our approach to be tailored to its unique setting and research aim.

Several challenges remained. First, despite our efforts to include a diverse group of PPCs, we were unable to recruit PPCs from lower- and middle-income countries. In future projects, we would like to develop strategies to foster greater diversity among PPCs, especially in terms of geographic location. Future projects might also aim to include a larger number of PPCs, depending on their needs. We realize that reporting guidelines are niche. Being involved as a patient or public member in the development of a reporting guideline may not be for everyone. It requires either a certain level of expertise or interest in learning about complex concepts, especially if it concerns a reporting guideline with a methodological focus like PRISMA-COSMIN for OMIs. One way to involve larger numbers of patients and public members might be to offer a parallel Delphi survey that focuses solely on candidate reporting items that matter most to them. This would require less information on technical concepts, and would reduce the participation burden on patients and public members. Second, we elected to let the patient partner participate in the Delphi study and therefore did not consult this patient partner when piloting the Delphi surveys. In future, we would definitely pilot all Delphi surveys with the patient partner to ensure the objectives, methods, instructions, and wording of items are understandable, regardless of participation in the Delphi study. Third, we did not capture prospectively what the impact of PPI was at each stage of the project; instead, we conducted retrospective evaluations, which may have resulted in missing an instance where PPI changed the content of the guideline. For example, we did not record how many times “tracked-changes” comments from PPIs as reviewers on various project documents were incorporated in the final deliverables. In the future, we recommend capturing changes resulting from PPI input in real time. Finally, even though we carefully planned and evaluated the PPI component of the project [31], we found that the content of existing evaluation tools was often not applicable to our project [22–25]. Therefore, informal feedback was solicited from the project team and the PPCs, instead of using a formal evaluation tool. Had evaluation tools designed for PPI in methodological research existed, it might had provided us with more comprehensive information about how to improve our methods throughout the project and in the future.

## Conclusions

We describe our approach to PPI in the development of a reporting guideline and share the lessons learned to encourage future guideline developers to integrate PPI in their reporting guidelines from a more informed position. Our approach to PPI in the development of PRISMA-COSMIN for OMIs was found to be feasible and considered valuable by PPCs and the research team. The “added value” of including PPCs in the development of PRISMA-COSMIN for OMIs was evident, as three items were included that might otherwise have been disregarded, and their suggestions for rewording made the guideline much clearer. The PPCs were keen to contribute to a project which ultimately impact patients, even though the scope of work is likely to be distant from PPCs’ immediate lived experiences. This blueprint was felt to be an important deliverable, as it shows how PPI can positively impact reporting guidelines. To user test the blueprint, we will contact researchers who register their intent to develop a reporting guideline on the EQUATOR website. This opens the possibility to receive feedback on how well the recommendations can be applied, and potentially increases the number of reporting guidelines that will integrate PPI as part of their development.

### Supplementary Information

**Additional file 1**. Feedback from the evaluation surveys administered after the onboarding session and Delphi study.

**Additional file 2**. Heatmap of evaluation surveys’ quantitative results.

## Data Availability

The datasets used and/or analysed during the current study are available from the corresponding author on reasonable request.

## Abbreviations

CIHR: Canadian institutes of health research
COSMIN: COnsensus-based standards for the selection of health measurement INstruments
E&E: Explanation & elaboration
EQUATOR: Enhancing the QUAlity and transparency of health research
GRIPP: Guidance for reporting involvement of patients and the public
OMI: Outcome measurement instrument
PEIRS: Patient engagement in research scale
PPC: Patient and public contributor
PPEET: Public and patient engagement evaluation tool
PPI: Patient and public involvement
PRISMA: Preferred reporting items for systematic reviews and meta-analyses
SPOR: Strategy for patient-oriented research
