# Supplementary material for: A blueprint for patient and public involvement in the development of a reporting guideline for systematic reviews of outcome measurement instruments: PRISMA-COSMIN for OMIs 2024
Source: Res Involv Engagem. 2024 Mar 21;10:33. doi: 10.1186/s40900-024-00563-5 (PMC10956212; doi:10.1186/s40900-024-00563-5)
Supplement: Supplementary file 1 — Additional file 1. Feedback from the evaluation surveys administered after the onboarding session and Delphi study. [file 40900_2024_563_MOESM1_ESM.docx]

**Additional file 1.** Feedback from the evaluation surveys administered after the onboarding session and Delphi study

| **Feedback after the onboarding session from evaluation surveys** |
| --- |
| General comments:   - I felt the onboarding made the work clearer to me, I now have to see the Delphi study​. - We will only really know how useful/suitable/helpful the onboarding information was when we apply it, when we do round 1. Thereafter, perhaps you could ask these questions again? For now, my view is prospective and theoretical, not yet reflective and practical. ​ - I think it was very good. You also provided pre-reading on "What is a Delphi study". That was helpful. ​ - Was good, felt welcome. Didn’t feel I had to be an expert to be there and try the Delphi study. - ​ Appreciated getting the slides ahead of time​. - I thoroughly enjoyed it​. - Well done!   Strengths:   - Very nicely organised and clear, thank you. Having a patient representative present, i.e. Maureen, saying that she had had some doubts, learned by doing, etc., made it feel like a "safer" space and experience ahead. ​ - The step-by-step explanation of the process we would be involved in​. - Friendly, supportive tone. Enough time for questions. The pause in the middle for questions was helpful. - Clarity and content.​ - Meeting the steering team, meeting other patients, explaining the slides in plain language, easy terms​.   Improvements:   - Would be nice if it’s in person​. - Nothing! - Nothing - Better able to assess this when I get the Delphi​. |
| **Feedback after the Delphi study from evaluation surveys** |
| - I found the estimates of how long it would take panelists--at least patient/public panelists--to complete each round were quite a bit shorter than the amount of time I actually spent. One result was that I often wondered if I was just too unfamiliar with everything about the project to be taking part in it. I probably spent at least three times the amount of time given in the various estimates, and even then, I sometimes felt as if I knew and understood too little to be a panelist. - Was a learning experience and I’m grateful. - There is a decided under representation of patient/public. I would encourage surveying a sizable number of patient stakeholders to see if this final version resonates with them, and if they even understand it or find value in it. - Thank you for making this process so clear, so well documented, and relatively accessible for patient and public participation. It is clear that a lot of reflection, planning, and good writing and editing have gone into it. - Whoever is responsible for all the support offered--the glossary, the summations of responses in earlier rounds, etc.--deserves much praise and thanks. |
