# Supplementary material for: A blueprint for patient and public involvement in the development of a reporting guideline for systematic reviews of outcome measurement instruments: PRISMA-COSMIN for OMIs 2024
Source: Res Involv Engagem. 2024 Mar 21;10:33. doi: 10.1186/s40900-024-00563-5 (PMC10956212; doi:10.1186/s40900-024-00563-5)
Supplement: Supplementary file 2 — Additional file 2. Heatmap of evaluation surveys’ quantitative results. [file 40900_2024_563_MOESM2_ESM.docx]

**Additional file 2**: Heatmap of evaluation surveys’ quantitative results

| **Administered scale - Time in project** | **+++** | **++** | **+** | **+/–** | **–** | **– –** | **– – –** |
| --- | --- | --- | --- | --- | --- | --- | --- |
| **Modified Acceptability E-Scale - After onboarding (n=5)** |  |  |  |  |  |  |  |
| Overall satisfaction with onboarding | 5 |  |  |  |  |  |  |
| Easy to attend onboarding | 5 | 0 | 0 | 0 | 0 | 0 | 0 |
| Acceptable duration of onboarding | 5 |  |  |  |  |  |  |
| Helped in understanding project goals | 5 |  |  |  |  |  |  |
| Enjoyment of onboarding | 3 | 2 |  |  |  |  |  |
| Helped in preparing for Delphi | 3 | 2 |  |  |  |  |  |
| Understandable information provided | 2 | 3 |  |  |  |  |  |
| **Modified PPEET - After onboarding (n=5)** |  |  |  |  |  |  |  |
| Available support | 5 |  |  |  |  |  |  |
| Informed about role in project | 5 |  |  |  |  |  |  |
| Good use of time | 5 |  |  |  |  |  |  |
| Clear purpose of onboarding | 4 | 1 |  |  |  |  |  |
| **Modified Acceptability E-Scale - After Delphi study (n=6)** |  |  |  |  |  |  |  |
| Onboarding helped in preparing for Delphi surveys | 4 | 1 |  |  |  | 1 |  |
| Delphi surveys helped in sharing thoughts and opinions | 3 | 1 |  | 2 |  |  |  |
| Acceptable duration of Delphi surveys | 3 | 1 |  | 1 |  | 1 |  |
| Overall satisfaction with Delphi surveys | 2 | 2 |  | 1 |  | 1 |  |
| Enjoyment in Delphi surveys | 1 | 2 |  | 2 |  |  | 1 |
| Ease of completing Delphi surveys | 1 |  |  | 2 |  | 3 |  |
| Understandable content Delphi surveys | 1 | 1 |  | 1 |  | 3 |  |
| **Modified PEIRS - After Delphi study (n=6)** |  |  |  |  |  |  |  |
| Introduced to research team | 5 | 1 |  |  |  |  |  |
| Clear communication research team | 5 | 1 |  |  |  |  |  |
| Addressing of concerns | 5 | 1 |  |  |  |  |  |
| Sufficient explanation Delphi study | 5 | 1 |  |  |  |  |  |
| Able to complete Delphi study | 5 | 1 |  |  |  |  |  |
| Able to provide perspectives | 5 | 1 |  |  |  |  |  |
| Received sufficient support | 5 | 1 |  |  |  |  |  |
| Sufficient time to complete tasks | 4 | 2 |  |  |  |  |  |
| Understood objective(s) Delphi study | 4 | 2 |  |  |  |  |  |
| Sufficient recognition for contributions | 4 | 2 |  |  |  |  |  |
| Good use of time | 4 | 1 |  | 1 |  |  |  |
| Manageable workload | 4 | 1 |  | 1 |  |  |  |
| Enjoyed Delphi study | 4 | 1 |  | 1 |  |  |  |
| Openness of research team to views | 4 |  |  | 1 |  | 1 |  |
| Worthwhile to complete Delphi study | 3 | 2 |  | 1 |  |  |  |
| **Modified PANELVIEW - After Delphi study (n=6)** |  |  |  |  |  |  |  |
| Appropriate consideration to patients' views | 5 |  |  |  |  |  | 1 |
| Involvement of key stakeholders | 4 | 2 |  |  |  |  |  |
| Transparency in feedback and changes | 4 | 2 |  |  |  |  |  |
| Clarity on final scope of guideline | 4 | 1 | 1 |  |  |  |  |
| Appropriate method to formulate reporting items | 3 | 3 |  |  |  |  |  |
| Transparent summary of evidence available | 3 | 2 |  | 1 |  |  |  |
| Participate again in Delphi study | 3 | 2 |  |  | 1 |  |  |
| Adequate time to complete tasks | 3 | 2 |  |  |  |  | 1 |
| Research team provided guidance during Delphi study | 3 | 1 | 1 | 1 |  |  |  |
| Research team managed group process | 3 | 1 | 1 | 1 |  |  |  |
| Appropriate consideration to external factors | 3 | 1 | 1 | 1 |  |  |  |
| Appropriate logistical support | 3 | 1 | 1 |  |  |  | 1 |
| Clearly defined objectives Delphi study | 3 |  | 1 |  | 1 |  | 1 |
| Information about methodology | 3 |  | 1 |  | 1 |  | 1 |
| Appropriate consensus method used | 2 | 4 |  |  |  |  |  |
| Appropriate method for decision-making | 2 | 3 |  | 1 |  |  |  |
| Overall satisfaction Delphi study | 2 | 3 |  |  | 1 |  |  |
| Appropriate consideration of evidence | 2 | 2 | 1 | 1 |  |  |  |
| Adequate preparatory work before Delphi study | 2 | 2 | 1 |  |  |  | 1 |
| Appropriate panel size | 2 | 2 | 1 | 1 |  |  |  |
| Adequate representation of backgrounds | 2 | 2 |  | 1 |  |  | 1 |
| Rigorous evidence synthesis | 2 | 1 | 1 | 1 | 1 |  |  |
| Clarity of wording reporting items | 2 |  | 2 |  | 1 | 1 |  |
| Required commitment appropriate | 1 | 3 |  | 1 |  |  |  |

Evaluation surveys after *onboarding* were completed by patient and public contributors (n=5); Evaluation surveys after *the Delphi study* were completed by patient and public contributors (n=5) and the patient partner (n=1); Items have been abbreviated for clarity purposes; Response formats were summarized as very positive (+++), positive (++), somewhat positive (only for PANELVIEW, +), neutral (+/-), somewhat negative (only for PANELVIEW, –), negative (– –), and very negative (– – –)
